# Are ape gestures like words? Outstanding issues in detecting similarities and differences between human language and ape gesture

**DOI:** 10.1098/rstb.2021.0301

**Published:** 2022-09-26

**Authors:** Catherine Hobaiter, Kirsty E. Graham, Richard W. Byrne

**Affiliations:** Origins of Mind Group, School of Psychology and Neuroscience, University of St Andrews, St Andrews KY16 9JP, UK

**Keywords:** evolution of language, gesture, flexibility, intention, common ground

## Abstract

Opinion piece: ape gestures are made intentionally, inviting parallels with human language; but how similar are their gestures to words? Here we ask this in three ways, considering: flexibility and ambiguity, first- and second-order intentionality, and usage in interactive exchanges. Many gestures are used to achieve several, often very distinct, goals. Such apparent ambiguity in meaning is potentially disruptive for communication, but—as with human language—situational and interpersonal context may largely resolve the intended meaning. Our evidence for first-order intentional use of gesture is abundant, but how might we establish a case for the second-order intentional use critical to language? Finally, words are rarely used in tidy signal–response sequences but are exchanged in back-and-forth interaction. Do gestures share this property? In this paper, we examine these questions and set out ways in which they can be resolved, incorporating data from wild chimpanzees.

This article is part of the theme issue ‘Cognition, communication and social bonds in primates’.

## 1. Introduction

Language may be the most powerful social tool that we employ as a species—with it we can express any concept, old or new, that comes into our minds and share it with the minds of those around us. Today we use it to send people into space, to write poetry, to gossip and share cat memes. But what were the first words like? What did we need to use our signals for, that took us beyond the systems that many other species around us use today?

Our closest living relatives, non-human great apes, have striking similarities to us in body plan and social cognition. Many papers, including our own, have made a case that understanding ape communication helps in our understanding of the origins of human language [1–9], but have these claims been justified? Great apes produce their gestures intentionally, that is, toward a specific recipient with a particular goal in mind [10–15]. Similar first-order intentional use in other species' signals has now been described—but typically in one or two highly specified signals (e.g. in fish: [16]). It remains only in ape gesture that there is abundant evidence for intentional use across a large repertoire of signals used in every-day communication [11,17]. Moreover, ape repertoires of gestures form extensive, rich systems of intentional communication (80+ signals; [11]), permitting nuanced investigation and affording an ideal opportunity to ask the question: are ape gestures rather like human words, and if so, how?

Before sketching some of the interesting similarities between the two systems, which are potentially important as clues to the evolution of language, we should be clear that there are also fundamental differences that, to date, seem unlikely to be easily resolved. In all human languages, building from within a shared ‘species’ set of foundational elements (e.g. phonemes; [18]), morphological components are learned by imitation of others, and differences between languages do not relate to genetic differences between populations. By contrast, great apes are unable to learn new vocalizations by imitation, and while captive studies of ‘ape language’ have shown that apes have the potential to learn new gesture types from human carers, in their natural gesturing they do not [15]. Their available repertoires of gesture types are species-typical, with an extensive commonality of both form and meaning between repertoires across great ape species [11,19,20]. The huge lexicon of words in most languages are a result of the multiple levels of patterning in language: in spoken languages (learned) morphemes are built up from more basic components of (innate) phonemes, which in turn are composed of articulatory features determined by the anatomy of the speech system. Nothing like this has yet been found in the gestural or vocal repertoires of great apes; consequently, while we may be impressed if a great ape shows a repertoire of 80 gesture types, a typical fluent English speaker has a repertoire of up to 20 000 word families [21,22]. Moreover, we find no evidence for higher-order structuring that resembles syntax in ape gesture: proving the negative is always tricky, but many studies including our own have looked hard for evidence of any rudimentary syntax in gestural sequences and found little trace [2,23–25]. This difference is critical to the open-endedness of language, in which essentially anything that can be thought can be expressed, and the set of sentences available to any user is infinite for practical purposes. These are big differences, but language must have had its origins in the communication system of an ancestor shared with living non-human apes, and it is to their communication that we must turn for evidence of what those origins looked like.

## 2. Flexible and ambiguous meanings of words and gestures

Humans and other great apes communicate across multiple modalities. Language can be independent of the channel in which it is expressed—e.g. spoken, signed, or written—and varied in the modality in which information is encoded (e.g. visual information is critical to discriminating some speech sounds [26,27]). Language is also often expressed through the combination of multiple channels and modalities [28], and the eventual integrated study of great ape signals across modalities will likely prove essential to a more complete understanding of language origins [29]. Nevertheless, we remain a long way off from being able to properly do so (although cf. [30–33]). While comparative study of primate communication, like much of linguistics, has a vocal-auditory bias [29], the similarity of ape gesturing to human language has been noted since the earliest observational work with wild apes [34,35]; and indeed, great ape gestures themselves span multiple modalities [36]. Moreover, while other modalities, such as facial expression and vocalizations, provide rich information to ape recipients [31,32], there remains only limited evidence that ape signallers deploy them in a goal-directed manner (cf. [37,38]).

Several different kinds of gesture are known in human communication, but the gestures of apes correspond to only one of them. We often gesture when we talk or sign, and these co-speech and co-sign gestures are best analysed as part of a language system, absent in apes. Many human gestures are arbitrary, socially transmitted inventions, like thumbs-up sign or a salute, in which the same form may have very different meanings in different cultures [39]; ape gestures are found across populations, and even different species, with shared meanings and the basic forms in their repertoire appear innately acquired [11,19,40]. For a human homologue of great ape gesture, we would point instead to the pre-linguistic gesturing of young children, which seems to drop away once we acquire adult language [41]. Though note that some of the gestures we might think of as cultural and learned may reflect this early shared repertoire: the palm-flat, outstretched hand request to be given something, a beckon, or a ‘shoo’ away gesture for instance, are shared with all great apes [19].

The behavioural context in which animal signals are produced can illuminate their use, for instance in studies of primate vocal communication [42–44]. Where we can accurately describe the conditions that produce the signal, we are able to hypothesize its adaptive function, for example when different alarm calls are made in response to different types of predator. We can then test hypotheses in different ways, exposing conspecifics to the stimuli that trigger it (e.g. predator sounds or models: [45,46]), or investigating behavioural responses to the signal to establish that the information is indeed present in the signal itself [47].

Many primate vocal signals allow their audiences to learn highly specific information, including the signaller's activity and identity [48–51], their emotional state [52], the nearby presence of a dangerous predator (functional reference, e.g. vervet monkey alarm calls; [45,53]), the dominance relationship between two individuals (e.g. in chimpanzee pant-hoots; [54,55]) or the quality of a food source (e.g. chimpanzee food grunts; [56]). Given the extensive repertoires and intentional nature of ape gestures, one might expect these gestures to contain similar or even greater specificity in information conveyed, so it may come as a surprise that gestures are used so flexibly across contexts and meanings [4,17,35].

A series of systematic studies across ape species, initially in captivity, highlighted the flexibility of ape gesture use. Individual gestures were found to be used across multiple behavioural contexts, and multiple gestures within the same context (e.g. food, travel, or play [7,10,13,17,57–59]). This so-called ‘means-end dissociation’ is a feature of language, and such flexibility in ape gestures suggested that they may be used in a more word-like fashion than many animal signals [35] which has inspired subsequent replication in wild populations (e.g. [4,19,20,25,40,60–63]). The case for flexible use of gestures was strengthened as researchers moved from a wide-angle view on the behavioural contexts in which gestures are deployed, to a more focused view identifying specific meanings associated with gesture types. Once again, many gesture types were associated with multiple meanings (e.g. come here, give me that, or let's travel), and meanings could be expressed by multiple gesture types [4,40].

Ambiguous meanings may seem like a problem for effective communication, but this is something that we readily overcome in human language. Exceptions such as onomatopoeia, highlight the apparently arbitrary relationship between spoken (and signed) words and their meanings, although there is increasing recognition that the arbitrariness of language may have been over-estimated [64,65]. Nevertheless, the meaning of a spoken word is not entirely specified by its form: the same articulation of sound or hand shape may be used for a range of different intended meanings, in obvious (‘bark’ tree/dog, ‘bank’ river/money), and subtle ways (‘nice shoes' may be a compliment, they are wearing amazing shoes, or a slight, the only positive you could find to say). Similarly in ape gesture, some gesture forms appear related to at least one of their meanings (for example, a *big loud scratch* or a *beckon*) but others do not (e.g. a *pirouette, arm raise,* or *object shake*). From the perspective of an effective and efficient system of communication, ambiguity in a signal's usage could be considered problematic, or at least potentially costly, so its widespread presence in a system is always intriguing. But the intent behind each of those ambiguous words is not ambiguous—the signaller has a specific meaning in mind—and in practice it usually works. While mistakes are sometimes made, most often the intent can be interpreted by the recipient, typically with no consciousness of any potential for other meanings [66]. One way in which we resolve the potential ambiguity is through physical and social context—are you at a garden centre or walking a dog? Talking about stocks or boats? In a pub or in an interview? The same may be true of ape gesture.

In the past, gesture researchers have tended to look for patterns of use across communicators, recipients, and contexts. However, in doing so they may have overlooked how socio-ecological features of the context of communication resolve apparent ambiguity. Recent research on bonobo gesture suggests that this may be the case [2]. Bonobo gestures, like those of other great apes, are usually ambiguous in the sense that a single gesture may correspond to several intended meanings. But the goal a bonobo signaller intended was found to be disambiguated almost completely by taking into account two simple aspects of the situational context of production: the activity in which the signaller was engaged, and its age and sex relative to the target audience [2].

So, is the same true of other apes? The answer appears to be yes. Here we provide a worked example of successful cases of gesture use in the Sonso East African chimpanzee community in the Budongo Forest, Uganda. Following Hobaiter & Byrne [4] play data were excluded, as gestures' use in play may not reliably signal gestural meaning outside of it. Once again, context—and to an extent signaller sex—appears to resolve apparent ambiguity in ape gestures. The use of *Big Loud Scratch* gestures, associated with two goals: Let's groom, and Follow me, is entirely disambiguated by Context alone (table 1). The gesture *Move Object* is more flexible, associated with four distinct goals (table 2), some of which appear initially contradictory: e.g. Move closer, and Move away. However, these two goals are again entirely distinguished by context. And while the gesture was most often recorded as used by male chimpanzees, female chimpanzee signallers were only recorded to use Move object gestures toward a single goal, Move away.

**Table 1.** Uses of Big Loud Scratch gesture by chimpanzees in the Sonso community distinguished by situational context.

goal	context	
	*grooming*	*travelling*
*let's groom*	✓	
*follow me*		✓

**Table 2.** Uses of Move Object gesture by chimpanzees in the Sonso community distinguished by situational context and by signaller sex (M = male, F = female).

	**context**										
	*all*	*agonistic*		*consortship*		*feeding*		*grooming*		*sex*	
**goal**		M	F	M	F	M	F	M	F	M	F
*follow me*	✓	✓		✓							
*move away*	✓	✓				✓	✓				
*move closer*	✓			✓				✓			
*sexual attention*	✓									✓	

Ape gestures thus appear to resemble words in both their flexibility and in the way in which the resulting potential ambiguity is resolved by context. Interestingly, this property suggests that rather than talking about ‘a’ gesture type used toward several meanings, we could consider each usage to be a separate gesture represented in output as a single physical form in the way that bark (tree) and bark (dog) are two distinct words that happen to be homophones. If this is the case, it suggests that we may have underestimated the number of ape gestures by a factor of 2–3, putting potential lexicons well into the hundreds.

So far, we have only addressed two aspects of context (behavioural and interpersonal); with sufficiently large datasets we may be able to add much more. One is the addition of other signals in different channels—for example: many gestures are associated with at least one ‘play’ meaning [4], including those that are used to address important non-playful goals, such as sexual solicitation or negation. Here, the simultaneous use of a ‘play face’ facial expression may disambiguate some gesture meaning [67], much in the way that facial expression can be used to indicate that someone is joking in spoken humour [68], or the addition of emojis to short-form text [69].

Aspects of individual identity or community membership have also been shown to shape ape social interactions in diverse ways [55,70–73]. There are already hints this may also be the case in gesture. In catalogues of ape gestural meanings, some goals were achieved by one or two gesture types, while others—in particular, negations—were associated with many more. One explanation [4] is that negations represent potentially costly requests, which may need to be finessed depending on who you are and whom you are interacting with: you might express a request for someone to leave the room quite differently when it is your boss rather than your little brother. We know relationship is important for humans; it may also be important for other great apes, yet this is an aspect of social context not captured by age and sex alone.

The next phase of sharpening our understanding of ape gesture meanings could therefore consider the extent to which the communicator is sensitive to broader elements of context with their recipient [74]. What if gestures, like words, are tuned to not only the immediate, but also the historic context of individual interactions? There is some evidence that selection of gestural signal modality is impacted by time previously spent in proximity with other individuals [75]. We might, for example, explore the possible impact not only of the signaller and recipient's individual social rank, but their relative rank to each other, established over previous interactions. Here we could explore the use of soft imperatives, or ‘polite forms’ of an imperative request. Importantly relative rank, unlike many characteristics of individual identity varies between two individuals across relatively short timespans – allowing for us to test change in gesturing within a particular pair of individuals, thereby controlling for other diverse aspects of social context. Here, for example, we could compare the use of common gestural requests between pairs of individuals before and after a rank reversal. While it is unlikely that in any one comparison it would be possible to control for many other aspects of social context that might vary, a consistent pattern of change across interactions may be suggestive of alternative forms, such as soft(er) imperatives.

Finally, some words remain highly specific, irrespective of context. It will be interesting to explore whether this is true of gesture, and if so whether there are particular categories of gesture types or gesture meanings that show little or no ambiguity, independently of context.

## 3. Let's move beyond imperatives; a declarative would be nice

Perhaps the most crucial limitation of current methods is an almost exclusive focus on imperatives, i.e. requests for the recipient to do something in response. Rather than necessarily a limitation of apes' gestural abilities, the restriction to imperative goals is a methodological limitation of the ‘Apparently Satisfactory Outcome’ approach [33,76,77]. Deducing intended meaning, unlike biological function, is not just a matter of ‘what happens next’ when a signal is given. Instead, we must work out which sequel is the one intended by the signaller. We do that by recording not only how the recipient changed their behaviour in response to the signal but also whether the signaller appeared satisfied with the response. Over many gesture instances of the same gesture type, we receive a distribution of apparently satisfactory behavioural responses for that gesture type (Apparently Satisfactory Outcomes, or ASOs), which can be used to infer the meaning.

Decoding gesture meaning in this way has been very productive. We have been able to describe the range of meanings for which great apes use their gestures across ape species [4,40,61,76,78], and found that both the physical form of the gestures in these repertoires [11,19] and the intended meanings for the apes [40] overlap extensively between species. East African chimpanzees and bonobos appear to use many of the same gestures for the same sets of meanings, although there are interesting differences in the prominence of certain meaning-gesture associations between the species [40].

But all these meanings are (necessarily, because of our discovery method) requests for the recipient to change their behaviour. While this method is an important tool for detecting specific meanings, we cannot capture meanings that do not require a change in behaviour. Declaratives, i.e. signals that make a statement about something, would not be detected; there is simply no reliable behavioural outcome for ‘what a lovely fig tree’ or ‘Alf is off in another party today’. Likewise, we would fail to detect requests to ‘keep doing exactly what you're doing’. One possible approach would be to measure recipient behaviour in more detail, for example measuring the duration of behaviour, as well as behavioural changes. In doing so it may be possible to show that a ‘keep grooming me’ gesture impacts the chances of grooming being stopped *in the near future*, even if there is no immediate change. If the apparent absence of ape gesture use for expressing declaratives is indeed an artefact of method, it is possible that their gestures are more deeply intentional than we realise. An alternative is that other types of meaning are truly absent from ape gesture—and demonstrating absence is always tricky—but given that we have only just started to find ways to explore declarative meanings, it would be very premature to exclude the possibility of more types of meaning.

## 4. Changing behaviour and changing minds: common ground with apes?

While there is substantial evidence for the intentional use of ape gesture, current evidence proves only first-order intentional use [12,79,80]. That is, the communicator recognizes that there is a distinct recipient and aims to change that individual's behaviour in line with a goal they want to achieve. But words are, at the very least, second-order intentional—when we use language, we recognize that the other individual is not only a distinct individual, but that they have their own knowledge, information, and goals. Is this a true difference between words and ape gestures, or a limitation on our ability to detect intentional use? The latter has been found in the study of Theory of Mind, where novel methods (e.g. [81,82]) steadily helped to reveal that at least some capacity to understand others' mental states (that I can imagine what you know, separately from what I know) is present in other species. If so—how do we move beyond current methods in gesture?

Intentions are not straightforward to explore; as properties of the underlying cognitive processes of the signaller, they are not something that observers have direct access to. Even with language, it is challenging to access intentions reliably in humans, and in other species we are even more dependent on interpreting patterns of observable behaviour [83]. In this regard, our methods have changed little since Darwin [84] and the early ethologists [85]. The communicator emits a signal, and the recipient interprets it [86]: this ‘behaviour-in, behaviour-out’ approach to interpreting nonhuman signals leaves cognitive process in a mysterious ‘black box’ [87]. In many ways this made substantial sense; our aim in studying nonhuman behaviour is to find the most parsimonious explanation. Many signals are broadcast, even if they are specific to a particular class of recipient (e.g. a specific species of pollinator (e.g. [88])), or a female at a particular stage of ovulation [89], and the communicator may have little or no voluntary control over how and when these signals are produced. Consider the changes in colour of an apple ripening or skin flushing, an odour given in response to fear or arousal, or an involuntary cry of alarm. And it is worth keeping in mind that we, with all our capacity for intentional language, also use broadcast signals.

However, the extent to which even broadcast signals are given may vary with socio-ecological context. Arousal effects, including audience effects, can lead to signals being more likely to be produced (e.g. [90]), or produced more often, for longer, or in the presence of stimuli such as individuals with whom we have a particular relationship (e.g. [91–93]). Expanding our methods to account for arousal is an important next step in assessing intentionality [94]. Communicators may have varying levels of voluntary control that allow them to suppress signals in particular situations, despite some of the necessary stimuli that trigger them being in place [95,96]. Again, human communication includes these same features: take our (sometimes failed) ability to suppress a smile or laugh that may be inappropriate in the moment.

But we—and some other species, including other apes—produce signals in another way too, one that cannot be described as broadcast. We choose to produce certain signals in a specific interaction, with a particular partner, to achieve a particular outcome. Here our audience is not just a class of recipient, or even just one specific recipient, but that individual at this moment in time, and our aim is to influence their behaviour in a particular way: through intentional communication. We know that in human communication with language, the aim is often more specific still: to influence the *mind* of a specific individual—‘second order’ intentional signalling. To describe intentional communication in the language-like sense, we must consider the shared knowledge space between signaller and recipient: their ‘common ground’. Rather than a solo performance on a stage, where we can consider first the communicator's then the recipient's perspectives, we have a pair of dancers, with the interpretation of the interaction only possible when the perspectives of both individuals are considered in tandem. Each communicator has (at least) the recipient's perspective in mind when they produce a signal (whether this be an audience of one or many).

Thus, in language, in addition to the signal and its context, the communicator's intention shapes the meaning for the recipient. A quick hello used with a nod of the head when two colleagues pass on the street (the context) could be both a polite greeting and a brusque dismissal depending on how the communicator intends for it to be understood. But, critically, the outcome of this interaction depends not only on the intention of the communicator but also on the understanding of it by the recipient. A key component in the intentional use of human language is that the communicator recognizes that, to achieve their goal, they must consider and adjust their words to take into account their recipient's state of mind. Second-order intentional communication is the recognition that not only is the recipient a distinct individual with their own perspective (first-order intentionality) but that they also have their own, potentially distinct, understanding and knowledge of the world [12].

Intentional communication has been argued to form the key cognitive distinction between language and other systems of communication [79,97], so when the case for first-order intentional use was established in ape gesture [13,17,98,99], it represented a substantial bridging of the gap between words and other species' signals. To date, evidence of the intentional use of ape gesture has been limited to first-order intentionality: the signaller recognizes that the recipient is a distinct agent and aims to shape their behaviour in a particular way. The next step, demonstrating the second-order use found in language, remains to be established.

There are already substantial hints, however, that apes have the capacity for second-order intentional communication. Work on the use of gesture by orangutans in interactions with their human caretakers suggested that they understood not only when their gestures had failed, but how their recipient may have misunderstood them in different ways [57]. In a request for a choice of food items, orangutans adjusted their subsequent gesturing depending on whether the recipient showed signs of incomplete understanding, by giving some but not all of the desirable food, or misunderstanding, by giving the undesirable food [57]. Similarly, chimpanzees producing a snake alarm call were sensitive not only to their relationship to the recipient, but also to whether the recipient was likely to already have the information about the threat [37,38].

While more formal tests of ape Theory of Mind have failed for many years (e.g. [100–102]), recent work employing eye-tracking, anticipatory looking, and violation of expectation paradigms suggest that their abilities may have been underestimated [82] although there remains substantial debate on this topic (cf. [103,104]). Similar technological developments may help in the detection of declarative meanings in gesture where the intention is to change something about the recipient's mind. The use of infrared thermography to detect subtle changes in physiological arousal not necessarily directly observable but linked to changes in, for example, emotion [105] has been successfully piloted with chimpanzee vocalizations [106] and may offer a new tool to access more subtle reactions. And, while the way in which we currently determine meaning is limited to gestures that are apparently successful, failure may be a productive means to investigate apes' understanding of other's minds, as shown in the orangutan study [57]. Where a gesture's meaning can be established from single, successful cases, we are able to then use this understanding to explore more complex interactions. If a signaller's initial gesture fails to achieve the intended outcome, do they persist with further gestures of the same type or elaborate with gestures of a different type; and crucially does their tendency to take one approach over another depend on the likely reason for the initial failure. A gestural request to a familiar partner, that has been successful many times before is probably not in this instance being misunderstood but is more likely a refusal. By contrast, the failure of a similar request to an unfamiliar partner, or one where the social relationship has recently changed, may be due to a miscommunication.

Gestures, like words, are intentional, but it remains to be discovered how intentional they are. As we consider intentionality and flexibility in relation to words and gestures, we must acknowledge that gestures and words are not only produced by someone—they are also interpreted by someone else and meaning can be formed through this interaction.

## 5. Words and gestures in conversation

In everyday interactions, language is not typically encapsulated in neat word(s) → response events. We exchange sentences and phrases back and forth in conversation, and even following imperative requests we often clarify and negotiate as well as act. However, despite widespread evidence for the presence of gestural exchanges in ape communication [25,107,108], these have only rarely been examined in terms of their relationship to language use. In most study of ape gesture, the signaller's goal has been considered at the level of the whole sequence (e.g. [4,40,57]). This is appropriately cautious: we only have one behavioural response that can be assigned to the point at which the signaller stops and appears to be satisfied. But in doing so we lose the opportunity to detect possible negotiation or changes of goal along a series of gestures, particularly when a gestural exchange takes place.

Gestural exchange could include a straightforward alternation of imperative demands, for example between two chimpanzee males of similar social rank—A: Groom me! B: No, you groom me. A: No, you groom me…—and so forth until one or both start to groom or both move on to other activities. In itself, this bears only limited resemblance to the syntax of exchanges in human conversation. But what if there is more going on? And how might we recognize it? For example, exchanges may also represent interactive negotiations, where the first signaller's original goal is shifted through ‘dialogue’ with the recipient. Or it may represent a confirmation of the signaller's request—A: Groom me! B: OK, I can groom you.

Recent work on turn-taking has started to incorporate a conversation-analytic approach to ape communication [109,110]; however, this has tended to focus on the exchange of behaviour such as gaze and response [60,108,111,112]. In one exception, the use of apparent exchanges in zoo-reared gorillas was described to incorporate negotiation of location and type of play [107], as well as negations within the exchange.

Established gesture meanings were used to explore possible changes in goal across a series of back-and-forth exchanges of gestures [107]. While this was a small preliminary dataset, there is substantial potential for developing this method with the much larger datasets available today and our understanding of how simple contextual information can disambiguate gesture meanings. The unambiguous meanings of these gesture–context pairings can then be used to analyse back-and-forth exchanges and may reveal the extent to which a signaller adjusts their initial communication in response to the recipient's. Declarative agreements might be trickier to establish. Exploring previously undetected changes in recipient behaviour with new tools, for example thermal imaging, or new uses of existing measures, may help. Particularly now that there is widespread strong evidence for the intentional nature of ape gestures, investigating those cases where response waiting (often a necessary marker of intentional use) is absent, may open up the range of meanings that can be detected, for example, a lack of response waiting may indicate that a response is not a second imperative request. Alternative tools might include thermal imaging, which was recently used to explore changes in arousal in wild chimpanzees' response to vocal signals [106] and during social feeding [113]. The combination of physiological measures with behavioural data on response-waiting or its absence may finally allow us to disentangle refusals or imperative demands to ‘do nothing’ from potential declaratives.

Expanding the timeframe in which we consider gestural exchanges beyond immediate gesture(s) → response may also be a fruitful line of enquiry. Take, for example, a chimpanzee consortship, in which a male and female chimpanzee move away from the rest of the group before she is at her ovulatory peak so that he can maintain exclusive sexual access to her through her fertile period [114]. These courtship solicitations can include intensive use of gestures [115,116], many of which are gesture types associated with sexual solicitation. However, the immediate goal (the outcome that stops the signaller signalling) is not sex but travel away. Copulation may occur hours or even days later. So, what is the male communicating? 'Follow me, now'; or 'Let's have sex, later'. Both are behavioural goals and both can be true, but in limiting the behavioural responses considered to those that stop the signaller from continuing to gesture in the short term, we necessarily exclude possible goals that occur over longer timeframes, or with intervening activities.

The interactivity of ape communication in general, and its similarity to language use, may only be fully assessed once we are better able to incorporate multi-channel analyses of their communication. Some characteristics of human language use, apparently absent in ape gesturing, are not typically expressed with words: signals of affirmation (yep, right, go on), or fillers that help establish common ground or repair miscommunication (hm, ah, huh, ahm). Detecting similarities and differences between ape communication and human language more holistically may allow us to better address the roles that words and gestures play within these systems.

## 6. Conclusion

A linguistic approach to non-human communication has been applied most extensively to semantics and syntax—what do signals look like, how are they ordered, and what do they mean? But, while an essential foundation, we are sceptical that this approach can alone fulfil the oft-promised contribution to the evolutionary origins of human language. When describing the distinction between an understanding of the properties of DNA and of the impact of our genes on behaviour, Sydney Brenner said ‘..the great difference between the telephone directory and a Shakespeare play is that, while both have a grand cast of characters, only the play has a plot.’ Our approach to exploring non-human communication to date gives us the tools in a species communication toolbox, it gives us the grand cast of characters, but what about the plot? It may seem like a theoretical stretch at times to ask – how is an ape gesture like a human word, not in its shape or structure, but in its use? But we suggest that this is the approach required to move forward in asking the questions that are key to understanding why human language emerged. Rather than, ‘What were the first words like?’, we suggest asking, ‘What were they used for?’ Here we have attempted to make a start on this enterprise, by going a little deeper into the use of ape gestural signals, asking to what extent they resemble words. We have compared human words with great ape gestures, proposing that both are used flexibly across multiple contexts; that both can be used for multiple meanings; that their meanings can be affected by context; that both are used intentionally; and that both are exchanged back and forth between individuals. Nevertheless, there remain important gaps to be addressed, particularly in terms of whether and to what extent gestures are used to address others' minds. Resolving these questions requires rethinking our approach to the exploration of non-human communication.

## Data Availability

This article has no additional data.
